# Beyond the radiology report: a multi-criteria decision analysis to define essential CT parameters for abdominal wall reconstruction: STAMP-C framework for preoperative hernia imaging

**DOI:** 10.1007/s10029-026-03706-7

**Published:** 2026-05-07

**Authors:** Fahim kanani, Narmin Zoabi, Benjamin T. Miller, Lucas R. A. Beffa, Clayton C. Petro, Ajita S. Prabhu, Guy Lahat, Eran Nizri, Yonatan Lessing, Adam Abu-Abeid, Michael J. Rosen, Nir Messer

**Affiliations:** 1 Department of Surgery, Gray Faculty of Medicine, Tel Aviv Sourasky Medical Center, Tel -Aviv University, Tel Aviv, Israel; 2 Department of General Surgery, Cleveland Clinic Center for Abdominal Core Health, Cleveland Clinic Foundation, Cleveland, OH USA; 3 Department of Gastroenterology, Gray Faculty of Medicine, Sheba Medical Center, Ramat Gan, Israel; 4 Department of Surgery, Gray Faculty of Medicine, Wolfson Medical Center, Holon, Israel; 5 Division of GI Surgery, Northwestern University and Feinberg School of Medicine, Chicago, IL USA

**Keywords:** Ventral hernia, Computed tomography, Standardized reporting, Multi-criteria decision analysis, Abdominal wall reconstruction

## Abstract

**Background:**

Comprehensive preoperative CT assessment is essential for ventral hernia repair, yet no standardized reporting framework exists. This study evaluated the completeness of preoperative abdominal CT reports and developed an evidence-based protocol to guide standardized reporting for abdominal wall reconstruction (AWR).

**Methods:**

We conducted a systematic evaluation of CT reporting completeness in 834 patients who underwent elective transversus abdominis release (TAR) at the Cleveland Clinic Center for Abdominal Core Health between January 2020 and December 2024. A panel of AWR experts defined 16 CT-based parameters deemed essential for surgical planning, and their clinical relevance was validated through a global survey of 61 AWR surgeons. Radiologic reports were assessed for documentation of these parameters and compared with intraoperative findings and registry data from the Abdominal Core Health Quality Collaborative (ACHQC). Parameters were classified as either generalizable or patient specific. A Multi-Criteria Decision Analysis using the Analytic Hierarchy Process was applied to prioritize features for standardized reporting.

**Results:**

Overall documentation completeness was limited, with a median of 34.4%. Although surgeons rated defect width as the most critical parameter for operative planning, it was documented in only 32.6% of CT reports. Patient-specific findings demonstrated higher overall reporting rates (median 87.8%), though key features such as mesh presence and anatomical mesh plane were documented in only 36.2% and 1.7% of applicable cases, respectively. Multi-Criteria Decision Analysis identified defect Size, Tanaka index, Anatomical hernia location, presence of prior Mesh, old mesh Plane and Concurrent inguinal or stomal site hernia as the most critical parameters for preoperative evaluation.

**Conclusion:**

Substantial gaps exist between CT reporting and the informational needs of AWR surgeons. We propose the “STAMP-C” framework as a pragmatic, consensus-driven model to standardize ventral hernia CT assessment and improve multidisciplinary alignment in preoperative planning. Prospective validation of this framework across diverse institutional settings and hernia subtypes is needed before universal adoption can be recommended.

**Supplementary Information:**

The online version contains supplementary material available at 10.1007/s10029-026-03706-7.

## Introduction

Preoperative planning for abdominal wall reconstruction (AWR) is inherently complex, necessitated by the challenges of reoperative anatomy, the heterogeneity of available surgical approaches, and the expanding spectrum of prosthetic mesh materials. As such, precise and comprehensive preoperative assessment is essential for optimizing operative outcomes [1]. Unlike small, primary ventral hernias, complex or recurrent defects often cannot be reliably characterized through physical examination alone [2]. Consequently, Preoperative imaging plays a pivotal role in procedural planning, with Computed tomography (CT) becoming the primary imaging modality due to its ability to delineate hernia morphology and assess critical anatomical relationships [3].

Despite its established role in surgical planning, a persistent discrepancy exists between radiologic reporting and the informational requirements of the operating surgeon. While this discrepancy has not been extensively characterized in the literature, Kushner et al. have shown that radiologic reports frequently omit critical findings essential to preoperative decision-making [4]. In our clinical experience, radiology reports from our institution and referring centers commonly fail to include fundamental hernia-specific features. These include parameters, such as identification of the previously implanted mesh plane and characterization of mesh type, and more readily straightforward features, including defect dimensions, retained foreign bodies (e.g., fixation devices), and calculation of the Tanaka index. Given the central role of preoperative imaging in planning ventral hernia repair, one would expect the existence of standardized reporting criteria similar to established reporting protocols in other areas of surgery. However, to date, no consensus exists regarding which parameters should be routinely included in CT reports for ventral hernia evaluation [5].

This study aims to systematically evaluate the extent to which radiologists document ventral hernia–specific findings in CT scans obtained for preoperative assessment and to identify potential targets for standardization in radiologic reporting.

## Methods

Following approval from the Cleveland Clinic Institutional Review Board, abdominopelvic CT reports were reviewed to assess the extent to which radiologists documented ventral hernia-specific features in adult patients scheduled for ventral hernia repair between January 2020 and December 2024 at the Cleveland Clinic Center for Abdominal Core Health (Cleveland, Ohio).

To enable in-depth analysis of reporting practices in complex scenarios, we deliberately selected a subset of patients who underwent elective, open Transversus Abdominis Release (TAR). Inclusion was restricted to cases in which CT scans were performed within six months before surgery and explicitly indicated ventral hernia repair as the intended procedure, ensuring that radiologists were aware of the surgical context. To further enrich the dataset with surgically meaningful findings, only patients with recurrent hernias previously managed with surgical intervention were included, allowing for assessment of whether prior repairs and implanted prosthetic materials were adequately documented in radiologic reports.

Eligible cases were identified through the Abdominal Core Health Quality Collaborative (ACHQC). The ACHQC is a hernia-specific nationwide registry aimed at improving the quality of hernia care through patient-centered data collection, performance feedback to clinicians, and collaborative learning Surgeons enter patient data prospectively in real-time during routine clinical care, including patient demographics, hernia characteristics, operative details, patient-reported outcomes (PROs), and postoperative follow-up information [6]. Once the relevant cohort was identified, corresponding CT reports were retrieved from the electronic medical record (EMR) using the Epic system. Only patients whose CT referrals met al.l predefined inclusion criteria were included in the final analysis.

To define the critical parameters for preoperative evaluation, we convened a panel of 12 AWR experts for structured discussion. The panel reached an expert consensus on a set of imaging features deemed essential for preoperative assessment and feasible to evaluate using CT. To supplement the expert input, a focused literature review was conducted to identify commonly cited CT-based parameters relevant to ventral hernia characterization and operative planning. The final list included 16 distinct radiologic features, each contributing unique and clinically significant information for surgical decision-making. These CT-based parameters were: defect width, defect length, number of defects, European Hernia Society classification for hernia location [7], Tanaka index [8], hernia content, presence of prior mesh, old mesh plane, central mesh fracture, mesh migration, presence of concurrent inguinal hernias, presence of concurrent parastomal or old stomal site hernias, abdominal wall anatomical abnormalities [9], rectus muscle measurements, patient habitus, and ongoing surgical site occurrences (SSOs). Further details regarding each CT-based parameter, including definitions, are provided in Table 1.

**Table 1:** CT-based parameters for planning AWR surgery

#	CT-based parameters	Definitions	AWR surgeons’ rating (Mean ± SD)
1	Defect width	Measured as the transverse distance between the medial fascial edges at the widest point of the hernia defect on axial CT view	9.16 ± 2.02
2	Concurrent parastomal or old stomal site hernias	Presence of a hernia adjacent to a stoma	8.93 ± 1.91
3	Old mesh plane	Determination of the anatomical layer in which the mesh is located (onlay, sublay, and underlay)	8.80 ± 2.25
4	Presence of prior mesh	Documentation of whether previously placed prosthetic mesh is visualized within the abdominal wall or peritoneal cavity	8.61 ± 2.25
5	Concurrent inguinal hernias	Identification of ipsilateral or contralateral inguinal hernias	8.54 ± 2.18
6	Tanaka index	Calculated as the ratio of hernia sac volume to total peritoneal volume	8.45 ± 2.32
7	Hernia location (EHS classification)	Anatomical location of the defect categorized according to the EHS classification system	8.36 ± 2.28
8	Abdominal wall anatomical abnormalities	loss or scarring of native fascial planes—particularly the posterior rectus sheath—often due to prior surgeries or infection. It is characterized by the absence of reconstructable tissue layers, necessitating component separation or myofascial release, and frequently requires biosynthetic or staged repairs due to compromised tissue quality and contamination risk[9]	8.17 ± 2.31
9	Number of defects	Represents the total number of discrete fascial defects identified on axial and coronal CT views	8.05 ± 2.53
10	Rectus muscle measurements	Quantitative assessment of rectus abdominis muscle width (from medial to lateral) and depth (ventral to dorsal) in the widest part of the hernia as seen in axial view	7.98 ± 2.18
11	Patient habitus and fat distribution	Evaluation of the predominant adipose tissue distribution, visceral versus subcutaneous, typically inferred from skin-to-fascia and fascia-to-bowel wall distances	7.97 ± 2.54
12	Mesh migration	Displacement of mesh from its intended anatomical location, either partially or completely	7.95 ± 2.36
13	Defect length	Measured as the longitudinal distance from the most cephalad to the most caudal extent of the fascial defect on a sagittal CT view	7.66 ± 2.73
14	Central mesh fracture	Evidence of structural discontinuity, segmentation, or tearing of a previously placed mesh prosthesis	7.64 ± 2.43
15	Hernia content	Abdominal viscera in the hernia sac	7.28 ± 2.98
16	Ongoing surgical site occurrences	Presence of wound-related complications such as surgical site infections (SSIs, per CDC definition), seroma, hematoma, abdominal wall abscess or enterocutaneous fistula	7.28 ± 2.47

To validate the clinical relevance of the identified CT-based parameters, we distributed an anonymous electronic survey to AWR specialists worldwide, including North America (United States and Canada), Western Europe (Germany, United Kingdom, France, and Spain), Australia, and Asia (India and Israel). The survey was distributed electronically via professional society networks — including the Americas Hernia Society and European Hernia Society — and through direct outreach to AWR specialists across these regions. The survey presented the list of 16 parameters and asked respondents to rate the clinical importance of each parameter on a scale from 1 to 10. Participants were also given the opportunity to suggest additional parameters they considered critical for preoperative planning. As the survey was anonymous and distributed without a defined sampling frame, a formal response rate denominator was not available. The survey was designed to assess face validity of the identified parameter list across a geographically diverse specialist population and was not subjected to formal test-retest reliability assessment.

To compare radiologic documentation with actual clinical characteristics, we extracted patient-specific hernia data from the ACHQC database and reviewed operative reports for all included patients. To assess the completeness of radiologic documentation of the 16 CT-based parameters, CT reports were evaluated independently by three reviewers using a standardized data abstraction chart. Quality assurance was maintained through calibration sessions, random audit checks, and intra-rater reliability assessments. Discrepancies were resolved by consensus, with a fourth reviewer consulted as needed. Reviewers were blinded to surgical characteristics. For meaningful assessment of the radiologic documentation, CT reports were then compared to actual clinical data. The 16 CT-based parameters were divided into two categories: eight generalizable parameters, defined as imaging features that can be evaluated independently of patient-specific surgical history. These included hernia defect width, defect length, number of defects, EHS classification for hernia location, Tanaka index, hernia content, rectus muscle measurements, and patient habitus. Documentation of these parameters was assessed using a binary scoring system, recording whether each feature was explicitly addressed in the radiologic report (yes/no). The second category included eight patient-specific parameters reflecting individualized clinical contexts and prior interventions. These encompassed the presence of previously implanted mesh, old mesh plane, central mesh fracture, mesh migration, presence of concurrent inguinal hernias, presence of concurrent parastomal or old stomal site hernias, abdominal wall anatomical abnormalities and ongoing SSOs. Documentation of these parameters was assessed by cross-referencing with operative findings and prospectively entered clinical data from ACHQC.

To establish a standardized set of CT-based parameters for abdominal wall reconstruction, we employed a systematic Multi-Criteria Decision Analysis (MCDA) approach integrating three distinct data sources. First, a panel of 12 AWR specialists conducted an expert audit, ranking 16 critical features through structured consensus discussion. Second, we surveyed 61 AWR surgeons globally who rated each feature’s importance on a 10-point Likert scale via an anonymous electronic survey distributed through professional society networks and direct specialist outreach across North America, Western Europe, Australia, and Asia. Finally, a systematic review of 40 studies assessed the frequency of feature documentation in the literature on preoperative CT assessment in ventral hernia repair. Each data source was weighted according to its authority, relevance, and methodological strength using the Analytic Hierarchy Process (AHP). Pairwise comparisons between criteria were performed using Saaty’s 1–9 scale, yielding normalized weights of 40.6% for the systematic review, 32.3% for the expert audit, and 27.1% for the surgeon survey. Individual feature scores were calculated by combining normalized scores from each data source, multiplied by their respective weights. The consistency ratio (CR) of the AHP pairwise comparison matrix was 0.082, below the accepted threshold of 0.10, confirming acceptable internal coherence. This comprehensive approach yielded a final prioritization ranking that balances clinical expertise, real-world practice patterns, and literature evidence. Features were subsequently categorized into three tiers based on natural score clustering: Tier 1 (Essential, score ≥ 9.0), Tier 2 (Highly Recommended, score 6.0–9.0), and Tier 3 (Recommended, score < 6.0). Full details of the AHP weight derivation, data source scoring, and feature prioritization are provided in the Supplementary Methods.

Statistical analysis included descriptive statistics for hernia characteristics and assessment of inter-observer agreement using Cohen’s kappa coefficient. We used Chi-square analysis to identify statistically significant differences between radiologists’ reported intention to document features and actual documentation rates. All analyses used SPSS version 28.0 (IBM Corp., Armonk, NY), with statistical significance set at *P* < 0.05.

## Results

**Fig. 1:**
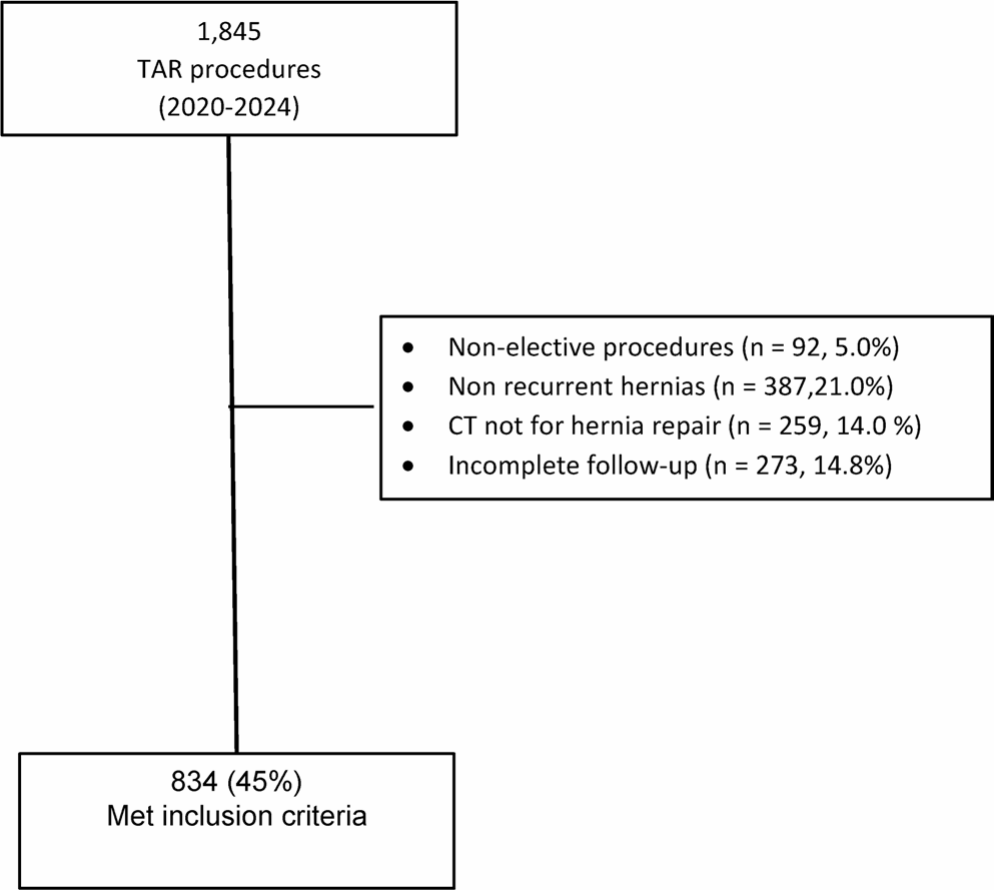
Flow chart of the inclusion criteria

Between January 2020 and December 2024, a total of 1,845 patients underwent elective TAR at the Cleveland Clinic Center for Abdominal Core Health. Of these, 834 patients met all predefined inclusion criteria (Fig. 1).

Baseline hernia and operative characteristics are summarized in Table 2. Among the 834 patients with recurrent hernias, 337 (40.4%) had undergone one prior repair and 240 (28.8%) had undergone two; the remainder had experienced multiple recurrences. Previously implanted mesh was encountered intraoperatively in 71.1% of cases (593/834). Among these, mesh was identified in the retromuscular plane in 821 patients (99.8%), with 516 (62.9%) also having preperitoneal mesh. Central mesh fracture was identified in 84 patients (10.1%), mesh migration in 77 patients (9.2%), and SSOs at the time of the index TAR procedure in 231 patients (27.7%). Concurrent stomas and inguinal hernias were each present in 34 patients (4.1%).

**Table 2:** Hernia and operative characteristics

Characteristic	*N* = 834
Hernia etiology	
Primary	0 (0%)
Incisional	834 (100%)
Hernia type	
Medline	762(91.4%)
Flank hernia	38(4.5%)
Parastomal hernia	34 (4.1%)
Number of prior hernia repairs	
One prior hernia repair	337 (40.4%)
Two prior hernia repairs	240 (28.8%)
Three prior hernia repairs	115 (13.8%)
Four prior hernia repairs	54 (6.5%)
Five prior hernia repairs	88 (10.6%)
Concurrent inguinal hernias	34 (4.1%)
Prior mesh found in the current repair	593 (71.1%)
Prior mesh plane	
Onlay	177 (30%)
Sublay	333 (56%)
Underlay	83 (14%)
Hernia length, cm (mean ± SD)	24.0 ± 6.2
Hernia width, cm (mean ± SD)	17.0 ± 6.8
Mesh-related complications	392 (47.0%)
Mesh fracture in previous repair	84 (10.1%)
Mesh migration in previous repair	77 (9.2%)
SSO found during the current repair	231 (27.7%)
Surgical site infection	46 (10.6%)
Seroma	25 (5.8%)
Hematoma	5 (1.2%)
Fistula	1 (0.2%)
Others	154 (18.5%)
Intraoperative complication	45(5.4%)
Bowel injury	16(1.9%)
Operative time>2 h	733(87.9%)

Radiologic documentation of the eight generalizable CT-based parameters is presented in Table 3. Defect width was reported in 272 reports (32.6%), defect length in 182 (21.8%), and number of defects in 262 (31.4%). Hernia location according to the EHS classification was reported in 222 cases (26.6%). The Tanaka index was documented in 108 reports (12.9%), and hernia content in 445 reports (53.4%). Rectus muscle measurements were documented in 3 cases (0.4%), and patient habitus in 2 cases (0.2%).

**Table 3:** Radiological documentation of eight generalizable CT-based parameters

Generalizable CT-based parameters	Marked in the CT report (%)
Defect width	32.6%
Defect length	21.8%
Number of defects	31.4%
Hernia location (EHS classification)	26.6%
Tanaka index	12.9%
Hernia content	53.4%
Rectus muscle measurements	0.4%
Patient habitus and fat distribution	0.2%

Documentation of the eight patient-specific CT-based parameters is shown in Table 4. Among the 593 patients (71.1%) in whom previously implanted mesh was confirmed intraoperatively, 215 cases (36.2%) included documentation of mesh presence in the CT report. The anatomical mesh plane was specified in 10 cases (1.7%). Central mesh fracture was found intraoperatively in 84 patients (10.1%), and reported radiologically in 87 cases, exceeding the number of confirmed intraoperative findings (103.6%). Similarly, mesh migration was identified in 77 patients (9.2%) and reported in 86 CT interpretations (111.7%). Concurrent inguinal hernias, parastomal or old stomal site hernias were each identified intraoperatively in 34 patients (4.1%). However, CT reports documented inguinal hernias in 82 cases (241.2%) and parastomal hernias in 80 cases (235.3%). Abdominal wall anatomical abnormalities were identified intraoperatively in 250 patients (30.0%) and reported in 180 cases (72%). SSOs were present in 231 patients (27.7%) and were documented radiologically in 51 cases (22.1%).

**Table 4:** Radiological documentation of eight patient-specific CT-based parameters

specific CT-based parameters	Occurrence (%)	Marked in the CT report	Marked in the CT report (% of Occurrence)
Presence of prior mesh	593 (71.1%)	215 (25.8%)	215 (36.2%)
Old mesh plane	593 (67.3%)	10 (1.2%)	10 (1.7%)
Central mesh fracture	84 (10.1%)	87 (10.4%)	87 (103.6%)
Mesh migration	77 (9.2%)	86 (10.3%)	86 (111.7%)
Concurrent inguinal hernias	34 (4.1%)	82 (9.8%)	82 (241.2%)
Concurrent parastomal or old stomal site hernias	34 (4.1%)	80 (9.6%)	80 (235.3%)
Abdominal wall anatomical abnormalities	250 (30%)	180 (21.6%)	180 (72%)
Ongoing surgical site occurrences	231 (27.7%)	51 (6.1%)	51 (22.1%)

A total of 61 AWR specialist surgeons completed the survey evaluating the perceived clinical relevance of the 16 defined CT-based parameters in the context of preoperative ventral hernia assessment. All parameters were considered clinically meaningful, each receiving a mean rating above 7 on a 10-point Likert scale. Defect width was rated highest (mean 9.16 ± 2.02), followed by presence of parastomal or old stomal site hernia (8.93 ± 1.91), anatomical mesh plane (8.80 ± 2.25), presence of prior mesh (8.61 ± 2.25), concurrent inguinal hernia (8.54 ± 2.18), Tanaka index (8.45 ± 2.32), EHS classification of hernia location (8.36 ± 2.28), and abdominal wall anatomical abnormalities (8.17 ± 2.31). The full ranking of all 16 parameters is shown in Table 1.

**Fig. 2:**
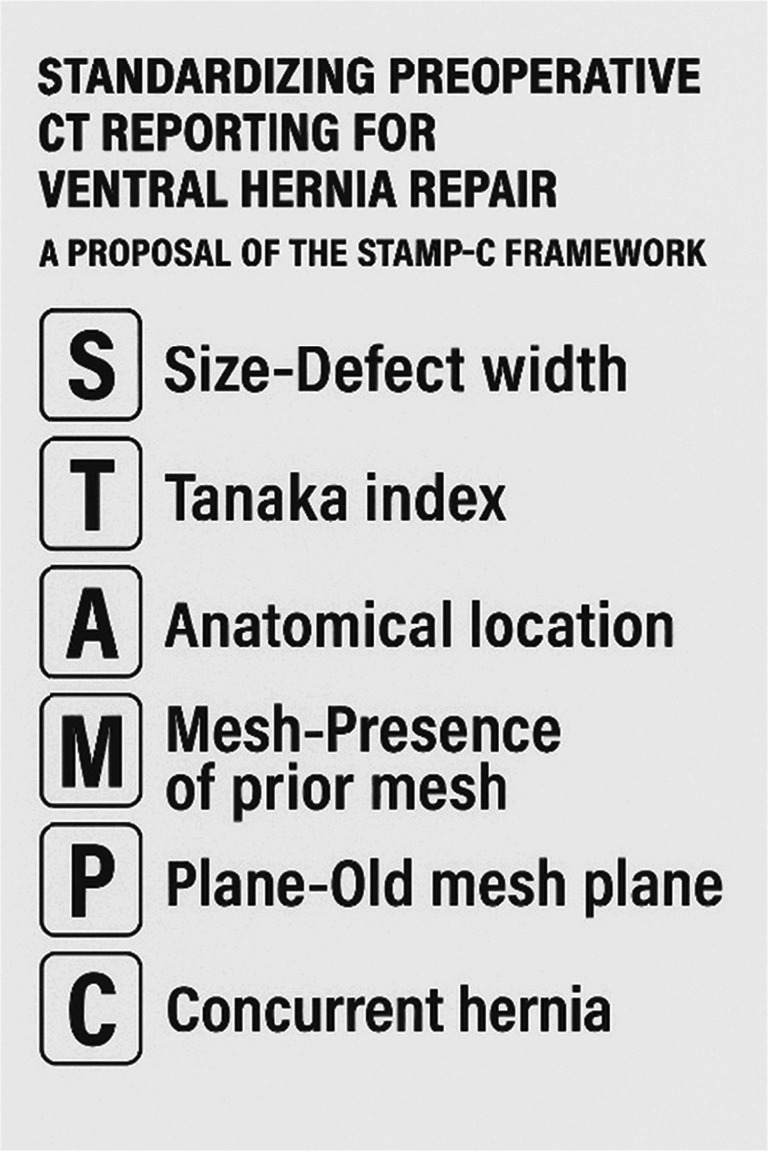
STAMP-C framework

Multi-Criteria Decision Analysis Outcomes are shown in Table 5. The systematic application of MCDA with AHP weighting yielded a comprehensive ranking of all 16 CT-based parameters that produced final scores that ranged from 14.51 (defect width) to 3.58 (Patient habitus and fat distribution). Tier 1 (score ≥ 9) included defect width, presence of prior mesh, old mesh plane, Tanaka index, hernia location and concurrent inguinal, parastomal and old stomal site hernias. Tier 2 (score 6.0–9.0) included anatomical abnormalities, rectus measurements and ongoing SSO. Tier 3 (score < 6.0) included the remaining six features. The top 7 parameters demonstrated consistent high ratings across all evaluative domains and achieved a MCDA score of 9 or higher. Notably, defect width score reflects its importance across all data sources: highest surgeon audit ranking (16/16), highest survey rating (9.16/10), most cited in literature (Fig. 2).

**Table 5:** Final Prioritization of CT-Based Parameters Using Multi-Criteria Decision Analysis

Rank	Parameter	Final Score	Tier	Recommendation
1	Defect width	14.51	1	Essential
2	Presence of prior mesh	13.21	1	Essential
3	Old mesh plane	12.15	1	Essential
4	Tanaka index	12.09	1	Essential
5	Hernia location (EHS)	11.13	1	Essential
6	Concurrent parastomal or old stomal site hernias	10.00	1	Essential
7	Concurrent inguinal hernias	9.52	1	Essential
8	Abdominal wall anatomical abnormalities	8.73	2	Highly Recommended
9	Rectus muscle measurements	7.58	2	Highly Recommended
10	Ongoing surgical site occurrences	6.94	2	Highly Recommended
11	Central mesh fracture	5.68	3	Recommended
12	Mesh migration	5.63	3	Recommended
13	Defect length	4.61	3	Recommended
14	Hernia content	4.60	3	Recommended
15	Patient habitus and fat distribution	4.28	3	Recommended
16	Number of defects	3.58	3	Recommended

## Discussion

Our findings reveal substantial discrepancies between the informational requirements of surgeons performing ventral hernia repair and the current standards of CT reporting. Overall documentation completeness was limited, with a median of 34.4%. Among the eight generalizable CT-based parameters, the median reporting rate was only 24.2% (range: 0.2%−53.4%). Patient-specific parameters demonstrated a higher median reporting rate of 87.8% when the condition was present (range: 1.7%−241.2%). While some parameters were reliably captured, such as central mesh fracture and mesh migration, which were documented at rates exceeding intraoperative detection (103.6% and 111.7%, respectively)—the general performance of CT reporting fell short of surgical expectations.

Notably, defect width—despite being rated as the most critical parameter—was documented in just 32.6% of reports. Parameters such as rectus muscle measurements and patient habitus, both critical for operative planning and mesh selection, were documented in only 0.4% and 0.2% of reports, respectively. In contrast to these generalizable parameters, patient-specific findings showed variable documentation rates. While mesh presence was documented in only 36.2% of cases where mesh was found intraoperatively, and the old mesh plane was specified in just 1.7% of cases, other patient-specific parameters like concurrent hernias were over-reported relative to their actual occurrence.

To our knowledge, this study represents the largest systematic evaluation of CT reporting practices for ventral hernia repair, encompassing 834 consecutive cases. Despite the critical importance of accurate and complete radiologic documentation in guiding preoperative strategy, most surgeons continue to rely on direct image interpretation rather than radiology reports [10]. This reliance reflects a significant gap in current practice, particularly in healthcare settings where surgeons may not have routine or immediate access to imaging platforms. In such contexts, comprehensive and standardized radiologic reporting is essential to ensure accurate preoperative assessment, facilitate operative planning, and support informed patient counselling.

Existing literature suggests that the deficiencies identified in our study reflect systemic rather than institution-specific issues in radiologic reporting practices. Halligan et al. [1, 11]

The need for standardized definitions in complex hernia care has been recognized beyond the domain of imaging. A recent Delphi consensus endorsed by the European Hernia Society established formal criteria for defining incisional hernias as ‘complex,’ underscoring the broader movement within the hernia surgery community toward consensus-driven standardization of clinical terminology and decision-making frameworks [REF]. The STAMP-C proposal aligns with this trajectory, extending the standardization agenda specifically to the radiologic domain — where, as our data demonstrate, the deficit is equally pronounced and the clinical consequences equally significant [12]. 

conducted a multicenter study across five European specialized hernia centers and found that only 12% of centers had established protocols for hernia-specific CT reporting, with 78% of surgeons routinely re-reviewing images due to inadequate radiologic documentation. Our deliberate inclusion of patients with recurrent ventral hernias undergoing open TAR permitted a focused evaluation in anatomically complex cases, where comprehensive and precise preoperative imaging is essential. Given the limited literature on this topic, the persistence of documentation gaps in our cohort—despite being drawn from a high-volume hernia center where radiologists routinely interpret imaging studies involving complex abdominal wall defects—underscores the broader relevance of our findings. These results suggest that the need for standardized CT reporting criteria in ventral hernia care is not confined to isolated practices but rather represents a widespread challenge across diverse clinical settings.

The reporting deficiencies identified in this study appear to exceed those documented in other surgical subspecialties. Nörenberg et al. [13] reported that despite standardized templates for rectal MRI, critical features were reported in only 38–52% of cases. Brook et al. [14] demonstrated similar gaps in pancreatic adenocarcinoma reporting, with resectability features documented in 45–51% of reports. In comparison, our findings demonstrate discrepancies of up to 70% between surgical findings and radiologic documentation, underscoring a more pronounced deficiency in the context of abdominal wall imaging. These findings suggest that the need for standardization in CT reporting may be particularly urgent in the evaluation of ventral hernias. The importance of standardized operative documentation has been similarly emphasized [15], and multidisciplinary team approaches have shown improved outcomes [16].

Several theoretical explanations may account for the observed discrepancies between the informational needs of surgeons and current CT reporting practices. One possibility is that radiologists may lack familiarity with certain hernia-specific parameters that are considered critical by AWR surgeons. Alternatively, some features may not be reliably discernible on CT imaging. Another contributing factor may be a limited awareness among radiologists of the clinical relevance of specific parameters that are considered essential by surgeons during operative planning. As this study was not designed to investigate the causes of these discrepancies, we are unable to definitively determine their origin. Nonetheless, to meaningfully improve reporting standards, we believe an important initial step would be the establishment of a clearly defined set of parameters considered essential by AWR surgeons for preoperative planning. The ultimate goal is to ensure that, in the event a patient presents with only a radiology report without access to the original CT images, surgical decision-making can still proceed without necessitating repeat imaging.

To prioritize the essential CT parameters out of the sixteen CT based parameters we applied a MCDA using the Analytic Hierarchy Process. Parameters with an MCDA score of ten or higher, including those in Tier 1 and the upper median of Tier 2, were identified as most critical, supporting their prioritization for standardized CT reporting. These parameters include defect width, presence of prior mesh, old mesh plane, Tanaka index, hernia location (EHS classification), concurrent inguinal hernia and concurrent parastomal or old stomal site hernia. In response, we modestly propose a pragmatic framework for abdominal wall imaging based on the mnemonic “STAMP-C”:

- Size - Defect width.
- Tanaka index.
- Anatomical location - Hernia location (EHS).
- Mesh - Presence of prior mesh.
- Plane - Old mesh plane.
- Concurrent hernia - Concurrent inguinal and parastomal or old stomal site hernia.

This study is subject to several limitations. First, the single-center design may limit the generalizability of the findings, although existing literature from other institutions supports the presence of similar deficiencies in radiologic reporting practices. Second, the use of operative documentation as the reference standard introduces the possibility of incomplete or inconsistent reporting. To mitigate this, we cross-referenced operative notes with data from the ACHQC registry to enhance accuracy and completeness. Nonetheless, intraoperative findings remain an imperfect reference standard. Certain CT parameters - including mesh plane identification, central mesh fracture, and mesh migration — may not be unambiguously resolved during surgery, particularly in the context of dense adhesions, prior component separation, or partial mesh incorporation. The over-reporting observed for parameters such as concurrent inguinal hernias (241.2% of intraoperatively confirmed cases) and mesh migration (111.7%) likely reflects radiologist over-calling of incidental or sub-threshold CT findings that do not meet the operative threshold for clinical significance, rather than systematic intraoperative under-detection. This discrepancy highlights that CT reporting for complex AWR requires not only completeness, but clinical calibration - a gap that the STAMP-C framework is specifically designed to address. Future studies should incorporate a composite reference standard combining dedicated radiologic re-read, operative findings, registry data, and postoperative imaging to more rigorously assess both reporting completeness and accuracy.

Importantly, some of the observed reporting deficiencies were not confined to surgeon-dependent findings. Generalizable CT-based parameters, those discernible independent of operative documentation, demonstrated similarly low rates of reporting.

Our use of a binary scoring approach, while effective for evaluating the presence or absence of documentation, did not assess the accuracy or qualitative adequacy of the reported findings. Reporting completeness and reporting accuracy represent two distinct dimensions of radiologic performance: a parameter may be documented but incorrect, or omitted despite being clearly identifiable on imaging. This study was designed to address the former - establishing how frequently surgically critical parameters are reported at all - as this constitutes the most fundamental and clinically actionable gap. Accuracy assessment would require dedicated radiologic re-read of all CT studies by subspecialty-trained abdominal radiologists, blinded to operative findings and applying pre-specified validated definitions for each parameter. The over-reporting signals identified in our data -particularly for concurrent inguinal hernias and mesh migration - provide a preliminary indication that documentation frequency alone does not guarantee clinical utility, and underscore the importance of a dedicated accuracy study as the necessary next step in this line of investigation.

The proposed set of essential parameters for preoperative planning was developed through expert consensus, an AWR surgeon survey, and informed by relevant literature; however, it has not been prospectively validated in a clinical trial. As such, its adoption should be approached with caution and adapted to local practice as appropriate. The MCDA prioritization framework employed the Analytic Hierarchy Process, a structured and validated methodology; however, the tier classification thresholds were derived empirically from natural score clustering rather than pre-specified a priori. While sensitivity analysis confirmed stability of the top-ranked parameters across variable weight distributions (see Supplementary Methods, Section 7), the tier boundaries themselves should be regarded as pragmatic organizational categories rather than validated decision thresholds. Independent replication using alternative weight configurations or additional data sources would strengthen the robustness of the final prioritization ranking.

The global surgeon survey was distributed via professional society networks and direct specialist outreach across North America, Western Europe, Australia, and Asia. As the survey was anonymous and distributed through multiple channels without a defined sampling frame, a formal response rate denominator was not available, and convenience sampling bias cannot be excluded. The survey was designed to assess face validity of the parameter list across a geographically diverse specialist population rather than to function as a psychometric instrument, and was not subjected to formal test-retest reliability assessment. The consistent agreement observed across all 16 parameters — each receiving a mean rating above 7.0 on a 10-point scale — supports the internal coherence of the expert panel’s judgments, though independent replication in a broader or more heterogeneous surgical community would further strengthen the generalizability of these findings.

A key methodological consideration is that this study was designed to evaluate radiologist reporting behavior, not to characterize the surgical patient population per se. The clinical cohort - patients undergoing elective TAR for recurrent incisional hernia with prior prosthetic mesh - was deliberately selected to create a standardized, high-demand CT context in which the indication for detailed reporting was unambiguous. All CTs were ordered explicitly for preoperative planning, with surgical intent clearly stated in the referral, thereby holding the informational expectations placed on the radiologist constant across cases and attributing reporting variation to radiologic practice rather than to differences in case complexity or referral quality. This selection was also intended to maximize the yield of patient-specific parameters - including mesh identification, mesh plane, and prior repair complications - which are precisely the features most consequential to operative decision-making in complex AWR. By interrogating radiologist performance in the most informationally demanding scenario, identified gaps carry direct clinical relevance. Nonetheless, documentation completeness rates observed here may not reflect reporting behavior for primary, non-recurrent, or laparoscopically planned hernias, in which the CT burden of information is lower and the clinical urgency of specific parameters may differ. The STAMP-C framework was developed from this specific context and should be prospectively validated across a broader range of hernia subtypes and institutional settings before universal adoption is recommended.

## Conclusion

This study demonstrates a substantial gap between the radiologic documentation provided in preoperative CT reports and the informational needs of surgeons performing abdominal wall reconstruction. By applying a structured methodology to prioritize essential radiologic features, we offer a core set of parameters deemed most critical for surgical planning. The proposed “STAMP-C” framework offers a practical, consensus-driven template that aligns with radiologic reporting norms while addressing surgical priorities. We encourage other institutions and professional societies to assess, refine, and validate this proposed framework in diverse clinical settings to promote broader standardization and enhance multidisciplinary collaboration in the care of patients with complex ventral hernias. While STAMP-C represents a rigorously derived, consensus-based proposal, it has not yet been prospectively validated. Its implementation should therefore be approached incrementally, adapted to local radiologic and surgical practice, and subjected to formal evaluation — including assessment of inter-radiologist reproducibility, impact on multidisciplinary communication, and effect on operative planning and patient outcomes.

## Electronic Supplementary Material

Below is the link to the electronic supplementary material.

Supplementary Material 1 (DOCX 41.8 KB)

## Declarations

### Registration of research studies

The World Medical Association's Declaration of Helsinki 2013 states in article 35: *'Every research study involving human subjects must be registered in a publicly accessible database before recruitment of the first subject'*. Editors require that all types of research studies involving human participants should be registered prospectively but failing that retrospectively. There are many places to register your research, and you can choose which is the most suitable for your needs:•https://www.clinicaltrials.gov/- for all human studies – free•http://www.chictr.org.cn/index.aspx- for all human studies – free•https://www.researchregistry.com/- for all human studies – charge•https://www.isrctn.com/- for all human studies – charge•Prospero- for systematic reviews – free•There are many national registries approved by the UN that can be found here We do not support or endorse any registry. Once registered, you will need to submit your assigned Unique Identifying Number (UIN) from your registration body as a mandatory part of your submission.

### Guarantor

The Guarantor is one or more individuals who take full responsibility for the work and/or the study's conduct, had access to the data, and had the final authority over the decision to publish Nir Messer.

## Abbreviations

AWR: Abdominal Wall Reconstruction
CT: Computed Tomography
TAR: Transversus Abdominis Release
EHS: European Hernia Society
SSI: Surgical Site Infection
ASA: American Society of Anesthesiologists
