# Supplementary material for: Beyond the radiology report: a multi-criteria decision analysis to define essential CT parameters for abdominal wall reconstruction: STAMP-C framework for preoperative hernia imaging
Source: Hernia. 2026 May 7;30(1):194. doi: 10.1007/s10029-026-03706-7 (PMC13152937; doi:10.1007/s10029-026-03706-7)
Supplement: Supplementary file 1 — Supplementary Material 1 (DOCX 41.8 KB) [file 10029_2026_3706_MOESM1_ESM.docx]

**Supplementary Methods: Multi-Criteria Decision Analysis for Standardization of CT-Based Hernia Assessment Parameters in Abdominal Wall Reconstruction**

**Overview**

To develop a standardized list of CT-based parameters for preoperative assessment in abdominal wall reconstruction (AWR), we employed a Multi-Criteria Decision Analysis (MCDA) approach. This methodology integrated multiple data sources and expert opinions to prioritize features based on their clinical importance and feasibility of implementation.

**1. Data Sources**

We collected data from three distinct sources, each contributing unique perspectives on parameter importance:

1. **Expert Surgeon Audit** (n=12 AWR specialists): Ranked 16 critical CT-based parameters through structured consensus discussion, assigning ordinal priority scores from 1–16
2. **Global Surgeon Survey** (n=61 attending surgeons): Rated 16 CT-based features independently on a 10-point Likert scale
3. **Systematic Literature Review** (40 studies): Assessed frequency of documentation and citation of each parameter across the published literature on preoperative CT assessment in ventral hernia repair

**2. Weight Assignment Using the Analytic Hierarchy Process (AHP)**

**2.1 Criteria Definition**

Three criteria were used to evaluate and weight each data source:

- **Authority**: Credibility and domain expertise of the information source
- **Relevance**: Direct applicability to AWR surgical planning
- **Methodological Strength**: Scientific rigor of data collection and analysis

**2.2 Pairwise Comparison Matrix**

Pairwise comparisons between criteria were performed using Saaty’s 1–9 scale:

| **Criteria** | **Authority** | **Relevance** | **Methodological Strength** |
| --- | --- | --- | --- |
| Authority | 1 | 3 | 1/5 |
| Relevance | 1/3 | 1 | 1/7 |
| Methodological Strength | 5 | 7 | 1 |

**2.3 Normalized Weights**

After matrix normalization and eigenvector calculation, the following criteria weights were derived:

- Authority: 0.196
- Relevance: 0.073
- Methodological Strength: 0.731

The consistency ratio (CR) of the pairwise comparison matrix was 0.082, below the accepted threshold of 0.10, confirming acceptable internal coherence of the weight assignments.

**3. Data Source Scoring**

Each of the three data sources was evaluated against the three AHP criteria using a 9-point scale, yielding the following scores:

| **Data Source** | **Authority** | **Relevance** | **Methodological Strength** |
| --- | --- | --- | --- |
| 12 Surgeons Audit | 7 | 9 | 7 |
| Surgeon Questionnaire (n=61) | 7 | 7 | 7 |
| Systematic Review | 9 | 9 | 9 |

**4. Total Score Calculation and Data Source Weighting**

Total scores for each data source were calculated using the formula:

**Total Score = (Authority Score × 0.196) + (Relevance Score × 0.073) + (Methodological Score × 0.731)**

Resulting data source scores:

1. Systematic Review: 9.000
2. 12 Surgeons Audit: 7.146
3. Surgeon Questionnaire: 6.000
4. Combined total: 22.146

These scores were normalized proportionally to derive the final weighting of each data source in the MCDA model:

- Systematic Review: 40.6% (9.000 ÷ 22.146)
- 12 Surgeons Audit: 32.3% (7.146 ÷ 22.146)
- Surgeon Questionnaire: 27.1% (6.000 ÷ 22.146)

**5. Feature Prioritization**

Individual CT-based parameters were scored by integrating normalized data from all three sources. Each parameter received a composite score reflecting its ranking across the expert audit, its mean Likert rating from the surgeon survey, and its frequency of citation in the literature, weighted according to the proportions derived in Section 4.

**Table S1. Final Prioritization of CT-Based Parameters**

| **Rank** | **Feature** | **Audit Score** | **Survey Rating** | **Literature Score** | **Final Score** |
| --- | --- | --- | --- | --- | --- |
| 1 | Defect width | 16 | 9.16 | 10 | 14.51 |
| 2 | Presence of prior mesh | 15 | 8.61 | 9 | 13.21 |
| 3 | Old mesh plane | 14 | 8.80 | 8 | 12.15 |
| 4 | Tanaka index | 13 | 8.45 | 9 | 12.09 |
| 5 | Hernia location (EHS) | 12 | 8.36 | 8 | 11.13 |
| 6 | Concurrent parastomal or old stomal site hernias | 11 | 8.93 | 7 | 10.00 |
| 7 | Concurrent inguinal hernias | 10 | 8.54 | 7 | 9.52 |
| 8 | Abdominal wall anatomical abnormalities | 9 | 8.17 | 7 | 8.73 |
| 9 | Rectus muscle measurements | 8 | 7.98 | 6 | 7.58 |
| 10 | Ongoing surgical site occurrences | 7 | 7.28 | 6 | 6.94 |
| 11 | Central mesh fracture | 6 | 7.64 | 5 | 5.63 |
| 12 | Mesh migration | 5 | 7.95 | 5 | 5.63 |
| 13 | Defect length | 4 | 7.66 | 6 | 5.68 |
| 14 | Hernia content | 3 | 7.28 | 5 | 4.61 |
| 15 | Number of defects | 1 | 8.05 | 5 | 4.28 |
| 16 | Patient habitus and fat distribution | 2 | 7.97 | 4 | 3.58 |

**6. Tier Classification**

Parameters were categorized into three tiers based on empirical score clustering of the final MCDA scores. Tier boundaries were not pre-specified a priori but were identified from natural breaks in the score distribution, and are consistent with the tier thresholds reported in Table 5 of the main manuscript:

- **Tier 1 — Essential** (Final Score ≥9.0): Defect width, presence of prior mesh, old mesh plane, Tanaka index, hernia location (EHS), concurrent parastomal or old stomal site hernias, concurrent inguinal hernias
- **Tier 2 — Highly Recommended** (Final Score 6.0–9.0): Abdominal wall anatomical abnormalities, rectus muscle measurements, ongoing surgical site occurrences
- **Tier 3 — Recommended** (Final Score <6.0): Central mesh fracture, mesh migration, defect length, hernia content, number of defects, patient habitus and fat distribution

**7. Sensitivity Analysis**

To assess the robustness of the final ranking, sensitivity analysis was performed by varying the normalized weights of the three data sources across a plausible range (±15% of assigned weight). The top seven parameters — defect width, presence of prior mesh, old mesh plane, Tanaka index, hernia location (EHS), concurrent parastomal or old stomal site hernias, and concurrent inguinal hernias — remained consistently ranked within Tier 1 across all tested weight configurations, supporting the stability of the essential parameter set.

**8. Limitations**

The MCDA model integrates three complementary data sources — expert consensus, global surgeon survey, and systematic literature review — but is subject to several constraints. The expert audit and surgeon survey reflect the perspectives of AWR specialists, which may not represent radiologic or generalist surgical viewpoints. The surgeon survey was distributed via professional networks, and a formal response rate denominator was not available; convenience sampling bias cannot be excluded. The literature review assessed documentation frequency as a proxy for parameter importance, which may not perfectly capture clinical utility. Tier classification thresholds were derived empirically from score clustering rather than validated against clinical outcome data. Future studies should prospectively assess the impact of implementing the STAMP-C framework on surgical planning quality and operative outcomes across diverse institutional settings.

**Supplements**

A) Surgeon Survey Instrument: CT Report of Ventral Hernia Adequacy (distributed via Google Forms to AWR specialists globally). Survey available at: https://docs.google.com/forms/d/e/1FAIpQLSc8NCdDXGLUwxQpWPd0q3p8OtDNP_Ehs31Gr6zzJORQmgBuQw/viewform?usp=header
